# Regional body composition and human core temperature responses to mild temperature water immersion in adults

**DOI:** 10.14814/phy2.70688

**Published:** 2025-12-09

**Authors:** Xiaojiang Xu, Stefan A. Gutierrez, Timothy P. Rioux, Scott J. Montain, David W. DeGroot, John W. Castellani

**Affiliations:** ^1^ Thermal and Mountain Medicine Division, U.S. Army Research Institute of Environmental Medicine Natick Massachusetts USA; ^2^ Oak Ridge Institute for Science and Education (ORISE) Oak Ridge Tennessee USA; ^3^ The Army Heat Center Fort Benning Georgia USA

**Keywords:** cold stress, hypothermia, regional fat mass, surface to mass ratio, thermoregulation

## Abstract

This project studied the effects of regional body composition on the core temperature responses to water immersion. Forty‐six volunteers participated in the study, with subgroups of eighteen immersed from the neck down in 18, 22, and 26°C water for up to 10 h, respectively. Regional body composition was measured by Dual‐energy X‐ray Absorptiometry. Rectal temperature (Tc), and 10‐site mean skin temperatures (Tsk) were measured every minute. Immersion durations ranged from 0.83 to 10 h, and Tc ranged from 35.2 to 38.0°C at the end of immersion. Tc cooling rates were calculated over the first 0.83 h of immersion. Tc cooling rates varied widely, ranging from −0.37 to 0.93°C/h, −0.39 to 1.87°C/h, and −0.13 to 1.13°C/h at 18, 22 and 26°C water, respectively. The trunk fat mass was negatively and significantly correlated to Tc cooling rates (−0.58, −0.76, 0.60, −0.64, *p* ≤ 0.01) at 18, 22, 26°C water and across all temperatures combined. The arm fat mass, fat percentage and surface‐to‐mass ratio were negatively and significantly correlated with Tc cooling rates at most conditions, but not all. Individuals with high cooling rates (≥0.6°C/h) had on average half the trunk fat mass of those with low cooling rates (≤0.25°C/h), and those with low trunk fat mass are least capable of defending core body temperature.

## 1 INTRODUCTION

Thermal and physiological responses to cold water exposure vary significantly among individuals. For example, during immersion in 10°C water, we observed that the body core temperature (Tc) of one volunteer dropped to 35.5°C after 20 min while other volunteers maintained their core temperature above 35.5°C over 120 min (Castellani et al., 2007; Xu et al., 2007). While physical properties such as the surface area‐to‐mass (SA/Mass) ratio (McArdle, Magel, Gergley, et al., 1984), subcutaneous fat thickness (Keatinge, 1960), lean body mass (McArdle, Magel, Gergley, et al., 1984), and total mass (White et al., 1992) have been shown to contribute to observed differences in responses, much of the variance has remained unexplained.

Body fat is a significant contributor to the relationship between human thermal equilibrium and tolerance to environmental cold stress (Hayward & Keatinge, 1981; Keatinge, 1960; Stocks et al., 2004; Toner & McArdle, 2011). It influences the physiological and internal thermal processes in two ways (Castellani et al., 2007): it provides insulation to the body and protection against Tc cooling and (Xu et al., 2007) it is typically associated with low metabolism and thus attenuates shivering heat production (Tikuisis et al., 1988). Most studies report an inverse relationship between body fat mass and Tc cooling rate (Hayward & Keatinge, 1981; McArdle, Magel, Spina, et al., 1984; Veicsteinas et al., 1982) although one study showed that subjects with low fat maintained a similar Tc, compared to their high fat counterparts, due to a significantly greater shivering thermogenesis during immersion in 18, 22 or 26°C water (Glickman‐Weiss et al., 1991). Some studies have suggested that the regional distribution of fat could be critical to thermal responses to cold (DeGroot et al., 2006; Diversi et al., 2016; Glickman‐Weiss et al., 1996).

The surface area‐to‐mass ratio (SA/Mass ratio) refers to the ratio between the surface area of an object and its mass. In the context of heat transfer, it is an index of how quickly an entire object can be cooled or heated. These properties led to the idea that the SA/Mass ratio might explain the individual differences in Tc responses during cold exposure. The results, however, have been mixed (DeGroot et al., 2006; Glickman‐Weiss et al., 1991; Hayward et al., 1975; Kollias et al., 1974; McArdle, Magel, Spina, et al., 1984; Shapiro et al., 1980; Taylor et al., 2024; Toner et al., 1986), with some studies reporting that a larger SA/Mass ratio resulted in greater decreases in Tc during water immersion (Kollias et al., 1974; McArdle, Magel, Gergley, et al., 1984) while others showed that differences in the SA/Mass ratio were unrelated to the Tc responses during cold water immersion (Glickman‐Weiss et al., 1993; Toner et al., 1986) and cold air exposure (DeGroot et al., 2006). One weakness of the SA/Mass ratio is that it ignores the large heterogeneity in body geometry within and between individuals.

We suspect that differences in regional mass and composition may contribute to the heterogenic response to cold exposure. It has been shown that spatial body temperature distribution and redistribution are dependent on body geometry and regional composition (Werner & Buse, 1988). Moreover, studies have revealed that regional fat insulation affects total body insulation (Veicsteinas et al., 1982) and Tc cooling rates are correlated with the fat thickness on both trunk and limbs while swimming in cold water (Sloan & Keatinge, 1973). Likewise, appendicular skeletal muscle mass on the arm and leg may also be predictive of the Tc response to cold (DeGroot et al., 2006). Therefore, the purpose of this study was to examine the relationship between regional body composition (e.g., fat, muscle mass) as determined by dual x‐ray absorptiometry (DEXA) and Tc cooling rate (°C/h) during immersion exposures in 18, 22 and 26°C water. The study aimed to determine whether regional body composition distributions are better predictors of human thermal responses to cold than fat% and SA/Mass ratio. We hypothesized that regional fat distribution would better predict core temperature cooling rates than lean body mass, percent body fat, and SA/Mass ratio.

## 2 METHODS

### 2.1 Volunteers

Forty‐six volunteers provided written, informed consent to participate in this study, which was approved by the US Navy Experimental Diving Unit (NEDU) Institutional Review Board. They volunteered after being fully informed of the requirements and risks associated with the research. The volunteers were 40 males and 6 females. Eight volunteers participated in two water temperature experiments while the rest participated in just one water temperature experiment. Each water temperature has 18 subjects. These data were collected as part of a project conducted to validate a human thermoregulatory model during prolonged immersion in warm water (Castellani et al., 2023).

### 2.2 Preliminary testing

All volunteers received a medical screening prior to participation. The volunteers then completed baseline testing which included nude height and body mass. Body surface area was computed from height and mass using the equation of DuBois and DuBois (Du & Du BOIS, 1916). Whole body and regional body composition were measured using a DEXA scanner (iDXA Machine, General Electric, Boston, Massachusetts). The lean mass includes all parts of the body (organs, muscle and fluids), excluding fat and bone mineral content (BMC). Table 1 provides the volunteers' physical characteristics grouped by water temperature.

**TABLE 1:** Physical characteristics of the subjects tested at each water temperature (mean ± SD, range, *n* = 18 at each water temperature).

Characteristic	18°C mean ± SD range		22°C mean ± SD range		26°C mean ± SD range	
Age (year)	35.9 ± 5.8	24.2–45.3	35.5 ± 8.1	23.2–55.4	38.8 ± 10.3	25.9–63.4
Height (m)	1.80 ± 0.07	1.65–1.9	1.77 ± 0.07	1.65–1.87	1.79 ± 0.06	1.67–1.89
Mass (kg)	93.1 ± 11.1	78.2–118.2	82.9 ± 13.2	56.1–105.2	88.9 ± 14.3	58.6–113.6
Fat% (%)	26.3 ± 7.3	11.9–37.5	25.3 ± 7.6	11.9–43	26.1 ± 8.6	8.7–37.7
SA (m^2^)	2.1 ± 0.14	1.91–2.38	2.0 ± 0.17	1.63–2.33	2.1 ± 0.18	1.66–2.34
BMI (kg·m^−2^)	28.8 ± 3.1	23.8–35.3	26.5 ± 3.8	19.97–30.97	27.6 ± 3.8	20.9–34.3
Female number	1		4		1	

### 2.3 Experimental protocol

The first step was a preimmersion medical screen. Then subjects were instructed to arrive at the testing site well hydrated and were asked not to consume alcohol for 24 h prior to immersion. Urine samples and preimmersion body mass measurements were taken after subjects were given a standardized meal based on approximately 33% of their estimated daily energy content, as estimated using the Harris‐Benedict equation multiplied by 1.4 for their physical activity level (Harris & Benedict, 1918).

The volunteers participated in one or two prolonged sustained immersions in water temperatures of 18 ± 1°C, 22 ± 1°C, and/or 26 ± 1°C. Each volunteer wore swim pants or a one‐piece swimsuit, and a type 1 life jacket. They were instructed to freely float in the water from the neck down. If any of the following events occurred, subject immersions were terminated: a volunteer requested termination, core temperature reached ≤35°C at any time or ≤35.5°C continuously for 5 min; a volunteer reached the maximum allowed time of 10 h immersed in water, or a research monitor deemed termination necessary for safety reasons.

### 2.4 Measurements

A rectal probe (YSI 400 series Xylem Inc., Rye Brook, New York) was used to measure rectal temperatures (Tc). The volunteers were given detailed instructions and then self‐inserted the rectal probe. The temperature probe was placed 15 cm from the probe tip to standardize the insertion depth. Volunteer skin temperatures were collected with a skin surface probe sensor (TE Connectivity, Schaffhausen, Switzerland) at 10 different sites (forehead, chest [pectoralis], abdomen, back [subscapular], upper arm [posterior], forearm [anterior], hand [dorsal], thigh [anterior], and foot [dorsal]). All temperature sensors were positioned on the right side of the body. Sensors were adhered to the skin using single‐sided waterproof medical tape. Rectal and skin temperatures were recorded every 30 s for 10 min before water immersion, during the immersion, and 10 min postimmersion. Environmental air temperature and relative humidity were measured above and adjacent to the pool where volunteers were immersed.

### 2.5 Post‐immersion

After immersion was completed, rectal and skin temperature data continued to be collected for 10 min. Soon after, volunteers were dried off, voided their bladders, and a post‐immersion nude weight measurement and a urine sample were taken. They were then seen and cleared by medical personnel, before exiting the testing area.

### 2.6 Statistical analysis

The Tc cooling rates were calculated as the Tc differences between the initial and 0.83 h (50 min) of immersion divided by 0.83 h to analyze the cooling rate and body characteristics. Correlation analyses (Pearson Product) were then used to compare the Tc cooling rate to the body characteristics, which included weight, height, regional body compositions (fat and muscle mass in the arms, legs and trunk), % body fat and SA/Mass ratio (GraphPad Prism, GraphPad Software, Boston, MA) within each cooling group and with the three groups combined.

## 3 RESULTS

Table 2 presents the regional fat, lean mass, and BMC in the arms, legs and trunk for each immersion group. Lean mass was the largest contributor to the total mass in each region and within each experimental group, and BMC was the smallest contributor.

**TABLE 2:** Regional fat mass, lean mass, and bone mineral content (kg).

	18°C		22°C		26°C	
	Mean	SD	Mean	SD	Mean	SD
Fat						
Arms	2.6	0.9	2.3	0.9	2.5	0.9
Legs	7.4	2.5	6.9	2.6	6.8	2.7
Trunk	13.9	5.3	11.1	4.8	13.6	6.5
LM						
Arms	8.8	1.6	7.5	2.1	8.4	1.8
Legs	22.7	2.8	20.3	3.9	21.4	3.2
Trunk	29.9	3.5	27.3	4	28.5	4.1
BMC						
Arms	0.5	0.1	0.4	0.1	0.5	0.1
Legs	1.3	0.2	1.2	0.2	1.3	0.2
Trunk	1	0.1	0.9	0.2	0.9	0.1

Abbreviations: BMC, bone mineral content; LM, lean mass.

There was large variation in immersion duration and Tc cooling rates irrespective of water temperature (see Tc responses of each volunteer in Appendix 1). In the 18°C water immersion group, the immersion durations ranged from 1.5 to 6.5 h. The initial Tc ranged from 36.8 to 38.0°C and Tc were 35.3 to 37.7°C at the end of immersion. The Tc of four volunteers dropped to 35.5°C or below and subsequently withdrew from the water, whereas the Tc of one volunteer increased after immersion into water and remained above their preimmersion Tc for about 2 h. At 22°C water temperature, the immersion durations ranged from 0.8 to 10 h. Initial Tc ranged from 36.8 to 37.7°C and Tc ranged from 35.4 to 38.0°C at the end of immersion. The Tc of two volunteers dropped to 35.5°C or below before exiting the water, and Tc of one volunteer increased after immersion into water and remained higher than the preimmersion Tc for about 1.5 h. At 26°C water temperature, the immersion durations ranged from 1.2 to 7 h. Initial Tc ranged from 36.6 to 38.2°C and Tc were from 35.4 to 37.2°C at the end of immersion. The Tc of only one volunteer dropped to 35.5°C or below before exiting the water.

**FIGURE 1:**
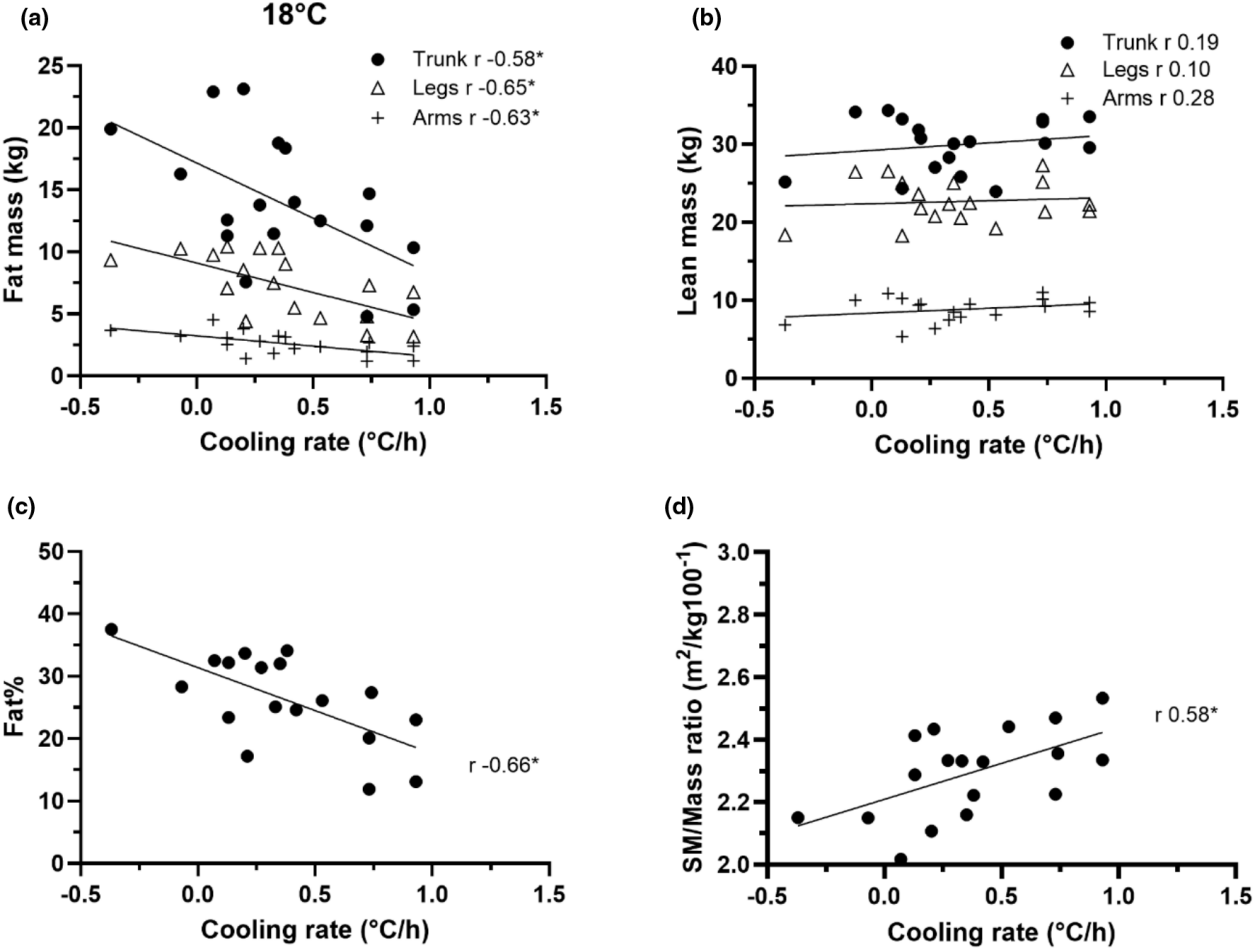
Scatterplots of regional fat mass in the arms, legs and trunk (a), lean mass in the arms, legs and trunk (b), fat% (c), and surface area to mass ratio (d) to absolute cooling rate in 18°C water.

**FIGURE 2:**
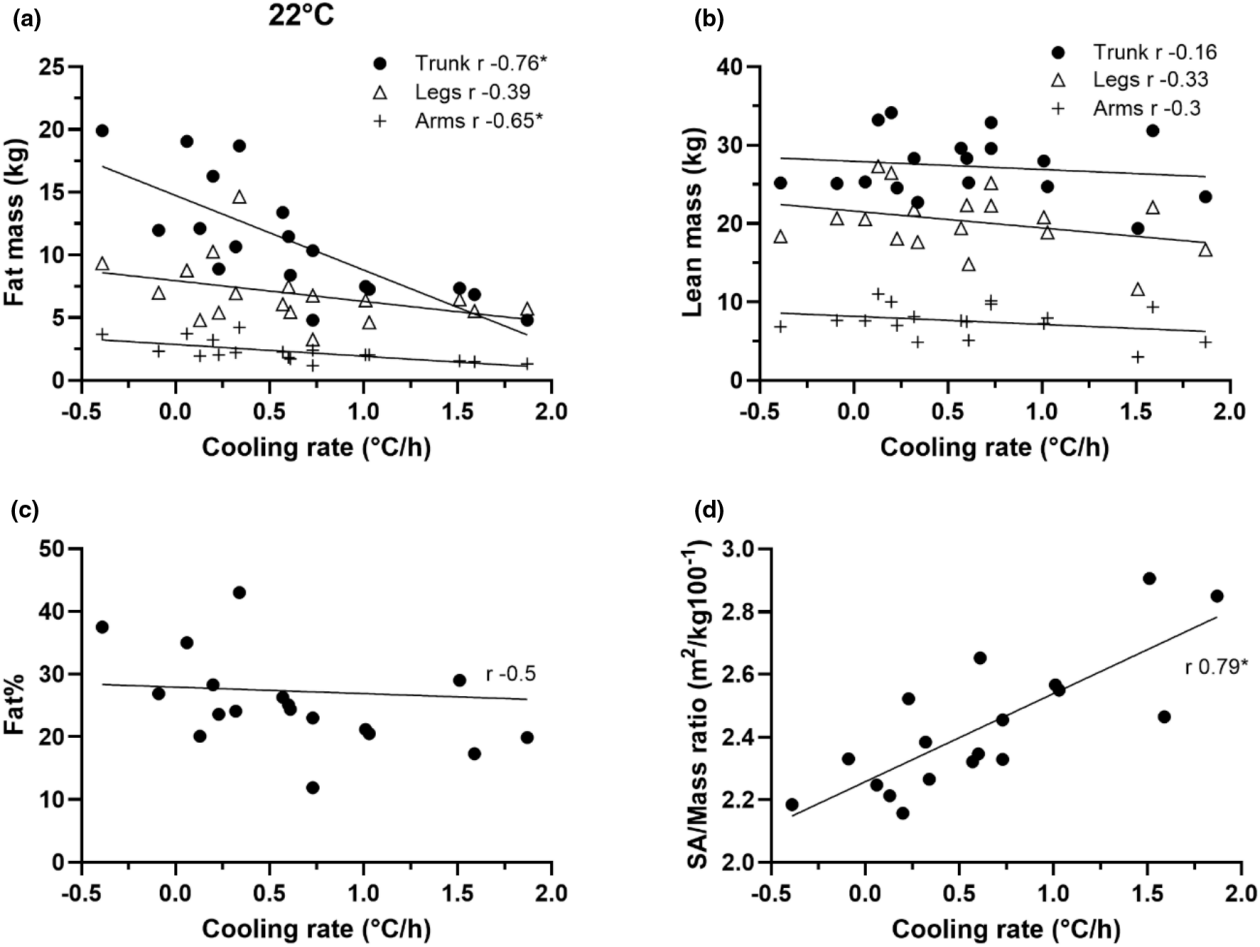
Scatterplots of regional fat mass in the arms, legs and trunk (A), lean mass in the arms, legs and trunk (B), fat% (C), and surface area to mass ratio (D) to absolute cooling rate in 22°C water.

**FIGURE 3:**
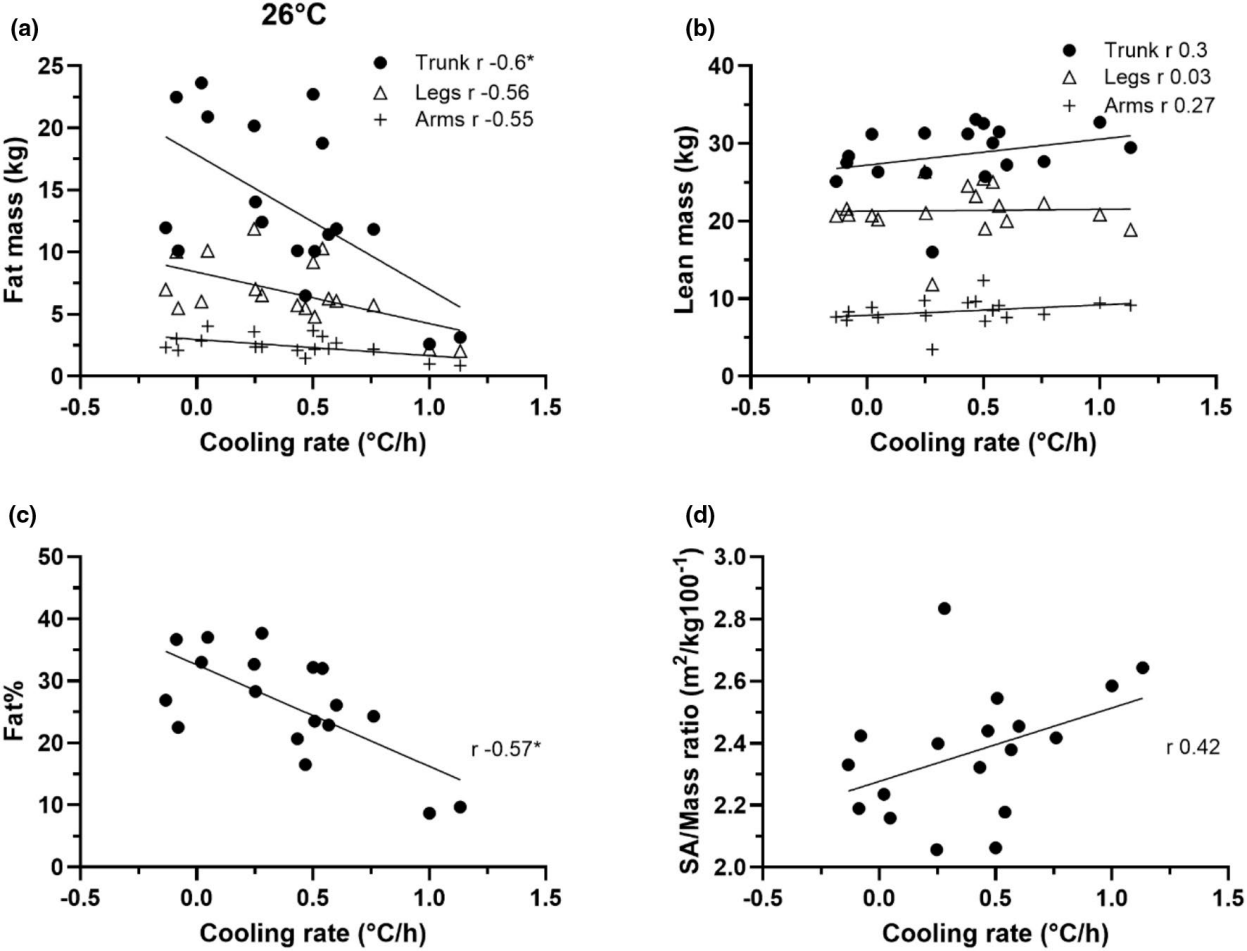
Scatterplots of regional fat mass in the arms, legs and trunk (a), lean mass in the arms, legs and trunk (b), fat% (c), and surface area to mass ratio (d) to absolute cooling rate in 26°C water.

Figures 1, 2, 3 present the relationships between regional fat or lean mass, whole body fat%, and the SA/Mass ratio with the Tc cooling rates during 18, 22 and 26°C water exposures. Table 3 presents the correlation coefficients (*r*) for these parameters, height, weight and Tc cooling rate. As illustrated in Figure 1A, in 18°C water, an inverse relationship between individual arm, leg and trunk fat mass and individual Tc cooling rates was observed, particularly between trunk fat mass and cooling rate. In contrast, there was no clear relationship between lean mass in the trunk, legs and arms and Tc cooling rate (Figure 1B). Figure 1C illustrates that there was a negative relationship between an individual's total fat% and their Tc cooling rate. Figure 1D illustrates that SA/Mass ratios ranged from 2.0 to 2.5 m^2^/kg·100^−1^. The Tc cooling rates, in general, increased as the fat% decreased and the SA/Mass ratio increased.

**TABLE 3:** Tc cooling rate correlation coefficients with body characteristics.

	18°C	22°C	26°C	All
Fat				
Arms	−0.63*	−0.65*	−0.55	−0.60*
Legs	−0.65*	−0.39	−0.56	−0.48*
Trunk	−0.58*	−0.76*	−0.60*	−0.64*
Lean mass				
Arms	0.28	−0.30	0.27	−0.09
Legs	0.10	−0.33	0.03	−0.20
Trunk	0.19	−0.16	0.30	−0.01
Fat%	−0.66*	−0.50	−0.69*	−0.57*
SA/Mass ratio	0.58*	0.79*	0.42	0.65*
Body Mass	−0.41	−0.63*	−0.32	−0.50*
Height	0.27	0.26	0.19	0.17

*p ≤ 0.01.

Consistent with the 18°C experiments, there was an inverse relationship between fat mass and Tc cooling rates (Figure 2A) during the 22°C exposures. The Tc cooling rates reduced as the trunk fat mass decreased from about 20 kg to 5 kg. The volunteer with the highest cooling rate had low trunk fat mass, 4.8 kg, but had around 20% body fat. The group's body fat ranged from 12 to 43%, and the SA/Mass ratio ranged from 2.2 to 2.9 m^2^/kg·100^−1^. The Tc cooling rates, in general, increased as the SA/Mass ratio increased.

As shown in Figure 3A, there was an inverse relationship between regional fat mass and Tc cooling rates. The groups fat% ranged from 9 to 38%, and the SA/Mass ratio ranged from 2.1 to 2.6 m^2^/kg·100^−1^. The Tc cooling rates increased as the fat% decreased. While increases in the SA/Mass ratio were related to the Tc cooling rate, there didn't appear to be any relationship between the 2.0–2.5 SA/Mass ratio.

The Pearson correlation coefficients for Tc cooling rate and several body composition parameters are presented in Table 3. The Tc cooling rates were negatively, consistently and significantly correlated with fat in the trunk (*p* < 0.01) at 18, 22, 26°C water temperatures and all water combined. The Tc cooling rates were also significantly correlated with the fat mass in the arms, fat %, and SA/Mass ratio at two water temperatures, but not at others.

**FIGURE 4:**
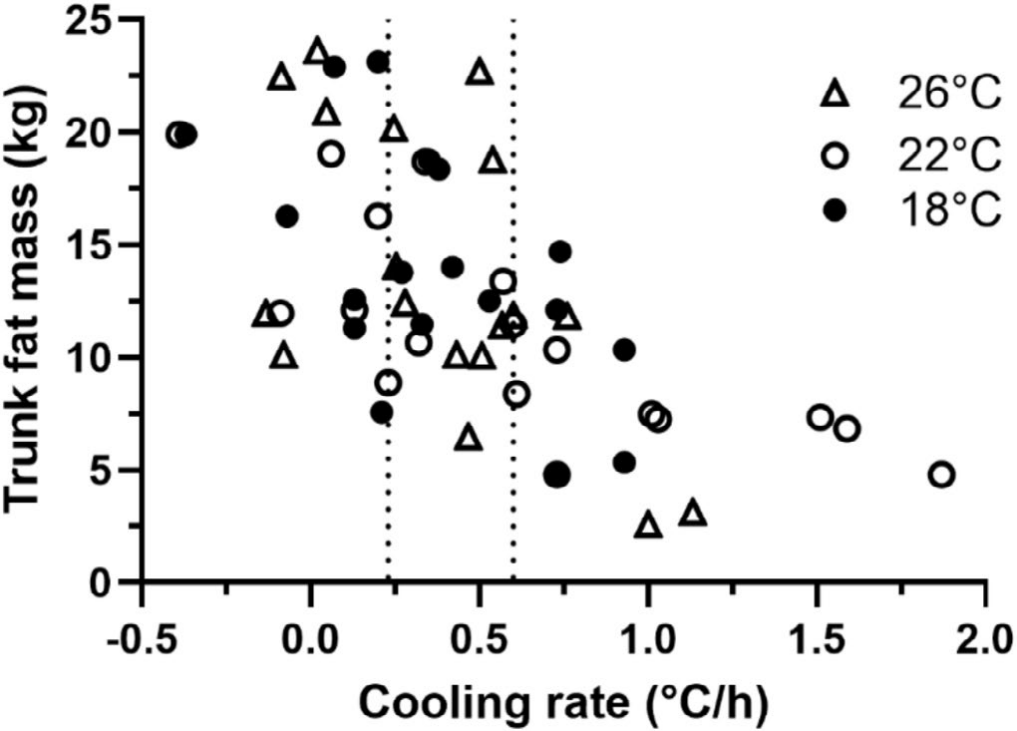
Scatterplots of trun fat mass and the Tc cooling rate during 18, 22 and 26°C immersion, high cooling rate ≥0.6°C/h and low cooling rate ≤0.247°C/h.

To further examine the relationship between the body composition and Tc cooling rate, data of the three immersion groups were combined, sorted by cooling rate, and further divided into three tertiles representing high, middle and low cooling rates. Figure 4 showed the trunk fat mass and the Tc cooling rates across all groups. The Tc cooling rates of each tertile were 0.6 to 1.86°C/h (high), 0.253 to 0.57°C/h (middle) and −0.39 to 0.247°C/h (low), respectively. Table 4 presents the body composition characteristics of the high and low cooling rate group. The water temperatures of each tertile were very close and thus the water temperature was not a factor which affects the Tc cooling rate. The average rates of the high and low Tc cooling groups were 0.78°C/h and 0.17°C/h respectively. The most substantial difference between the high and low cooling groups was the trunk fat mass. The average trunk fat mass of the high cooling rate group was 8.08 ± 3.5 kg while the average trunk fat mass of the low cooling rate group was 16.17 ± 5.4 kg.

**TABLE 4:** Body characteristics mean (SD) of high and low cooling rate groups.

	High cooling rate group	Low cool rate group
Cooling rate (°C/h)	0.78 (0.49)	0.17 (0.17)
Water temperature (°C)	21.78 (2.90)	21.56 (3.3)
Arm fat mass (kg)	1.79 (0.57)	2.97 (0.85)
Leg fat mass (kg)	5.17 (1.72)	8.00 (2.10)
Trunk fat mass (kg)	8.08 (3.50)	16.17 (5.43)
Fat%	19.81 (6.30)	29.57 (6.37)
SA/Mass ratio (m^2^/kg)	0.025 (0.002)	0.023 (0.001)
Body mass (kg)	79.82 (10.90)	94.61 (12.28)
Height (m)	1.79 (0.06)	1.77 (0.08)
Subject numbers	18	18

## 4 DISCUSSION

The aim of this project was to examine the relationships between an individual's body composition characteristics and their Tc cooling rates during immersion in 18, 22 and 26°C water. The major findings are that: (1) cooling rate is inversely and significantly related to trunk fat mass at 18, 22, and 26°C water and across three temperatures combined, (2) individuals in the high cooling rate group (≥0.6°C/h) have approximately half the trunk fat mass of those in the low cooling rate group (≤0.25°C/h), and those with low trunk fat mass are least capable of defending body core temperature, and (3) Tc cooling rate is related to the body composition characteristics, that is, total body fat% and surface area to mass ratio, but the relationship is only apparent in some cooling conditions.

A strength of this project is the broad range of fat mass in the subject population and the between subject heterogeneity in its distribution. Moreover, this heterogeneity was present within the groups tested at each water temperature. On average, the trunk fat mass was 1.8 times greater than the leg fat mass, ranging from 0.8 to 3.9 times. Likewise, the trunk fat mass was on average 5.1 times greater than the arm fat mass, and ranged from 2.6 to 8.3 times. As a result, the experiment was able to reveal that seven individuals with relatively low trunk fat (range 2.6 to 7.5 kg), and in whom the trunk fat mass was close to the leg fat mass (range 2.2 to 6.4 kg). All those individuals had Tc cooling rates more than 1.0°C/h, as shown in Figure 4. The individual volunteer whose Tc cooling rate was the highest, at 1.9°C/h, had only 4.8 kg fat in the trunk and 5.7 kg fat in their legs, as shown in Figure 2A. In contrast, as shown in Figure 1 and Figure 2A, the single volunteer who was capable of preserving the body core temperatures for more than 90 min (i.e., negative cooling rate, −0.37 and −0.29°C/h) at both 18 and 22°C immersion possessed one of largest trunk fat masses within the subject population studied, about 20 kg in trunk, and 9.3 and 2.7 kg fat mass in their legs and arms, respectively. Table 3 reveals that trunk fat mass is significantly correlated with Tc cooling rates across all conditions and is more predictive of Tc cooling rates than other parameters. Table 4 demonstrates that individuals with low amount of trunk fat, especially 8.1 kg or less, are least capable of defending body core temperature. Previous data suggested that regional body composition could be related to Tc responses to cold (DeGroot et al., 2006; Diversi et al., 2016). Our current study demonstrates that the trunk fat mass is inversely, consistently and significantly correlated with Tc cooling rate during water immersion in 18–26°C water.

The contributing role of fat mass and particularly trunk fat mass in the preservation of body heat during water immersion likely stems, at least in part, from its thermal properties. Adipose tissue has low thermal conductivity, which contributes to the preservation of body heat and defense of core temperature. Fat thermal conductivity is 0.21 W/m°C, which is lower than either skin (0.47 W/m°C) or muscle (0.51 W/m°C) (Werner & Buse, 1988) and is equivalent to thermal resistances of 0.048, 0.021 and 0.02 m^2^ C/W per 1 cm tissue for fat, skin and muscle respectively. During cold water exposures, 75% of the shivering heat production (increased metabolism) is generated in the trunk (Bell et al., 1992) and body core temperature (rectal, esophageal) is typically higher than the temperatures in legs and arms. For example, after immersion in 8°C water for 1 h, the deep tissue temperatures in the upper leg and lower leg were 0.7°C and 10°C below the Tc, which was about 34°C (Bristow et al., 1994). The trunk has been shown to be the main site of heat loss during rest in cold water (Hayward & Keatinge, 1981; Riera et al., 2014). Therefore, having a surplus of fat in the trunk (relative to legs or arms) could potentially support defense of body core temperature.

Fat% was inversely related to the Tc cooling rate, as shown in Figure 1, Figure 2, and Figure 3C. A weakness with using fat% as an index, however, is that it doesn't resolve differences in regional distribution of fat. As shown in Figure 2A,C (22°C immersion), the three volunteers with high Tc cooling rates of 1.4°C/h or more possessed 17%–29% fat, but they had relatively small amounts of fat in their trunks. The volunteer with 29% fat had 7.3 kg and 6.4 kg fat in their trunk and legs respectively. Likewise, the individual with 17% fat, possessed only 6.4 kg in their trunk and 5.5 kg in the legs. Lastly, the individual with 20% fat had 4.8 kg in their trunk and 5.7 kg of fat in their legs. This in part explains the results in Table 3 and Figure 2C that fat% is not significantly related to Tc cooling rates at 22°C condition. Therefore, while fat%, as a whole‐body index, is associated with the Tc responses to cold water immersion at most conditions, it is prone to error due to the heterogeneity of fat distribution across individuals.

The role of SA/Mass ratio in human thermal responses to cold has been investigated in numerous studies. From the perspective of heat transfer, a higher ratio means that there is more surface area available relative to mass, facilitating/allowing for faster heat loss. Some studies demonstrated that a larger SA/Mass ratio resulted in greater decreases in Tc during water immersion (Kollias et al., 1974; McArdle, Magel, Gergley, et al., 1984) while others showed that differences in SA/Mass ratio had minimal impact on Tc responses during water immersion (Glickman‐Weiss et al., 1993; Toner et al., 1986) and during cold air exposure (DeGroot et al., 2006). The inconsistent relationship was also present in the current study. For example, as shown in Figure 1 D, six volunteers with a similar ratio around 2.3 (m^2^/kg100^−1^) possessed Tc cooling rates that ranged from 0.15 to 0.85°C/h in the 18°C water condition. Conversely, in the 26°C water condition (Figure 3D), three volunteers with Tc cooling rates of about 0.25°C/h possessed SA/Mass ratios ranging from 2.1 to 2.8 (m^2^/kg100^−1^). Regional differences in SA/Mass ratios might explain part of the observed variance in cooling response. A separate study has reported that regional SA/Mass ratios of two adults ranged from about 1.8, 7.4 and 11.7 (m^2^/kg 100^−1^) for the torso, feet and hands, respectively while the whole‐body ratio was 2.9 (Xu et al., 2017). Thus, between‐subject variability in regional fat mass may explain why the SA/Mass ratio is proportional to Tc cooling rate in some immersion conditions but not in others.

The practical implication of this study is that the regional body composition affects human thermal responses to cold water immersion. Awareness of regional fat, or the lack thereof, in the trunk may be predictive of who will be more vulnerable to developing hypothermia. Hypothermia occurs during occupational or recreational activities. Tc may drop quickly and hypothermia risk is high when Soldiers cross rivers or swim in cold water during training or operations, Coast Guard staff rescue victims in water, and professional and nonprofessional swimmers swim in cold water for prolonged periods. Estimates of body composition, from DEXA (as in the present study) or other methods, can be used to assess the individual risk of hypothermia and better prepare for expected exposures and preventive measures. In addition, it is possible to use regional body composition to develop individualized hydrotherapy for athletes when body cooling rate is important for treatment purposes. Regional body composition provides a new perspective or a new approach to study individual thermal responses to heat and cold.

This study has limitations. The metabolic rates were not measured; thus, the effects of regional body composition on the shivering heat production could not be examined. In addition, 40 of the volunteers participated in a single trial, with 8 volunteers participating in 2 trials, increasing between‐trial variability. Thus, it was not possible to examine individual Tc responses as a function of water temperature. In addition, the water temperatures were mild and human thermal responses might be different at colder water temperatures.

## 5 CONCLUSIONS

This paper studied the effects of regional body composition determined via DEXA on the core temperature responses during immersion in 18, 22 and 26°C water. The trunk fat mass was negatively and significantly related to Tc cooling rates at 18, 22, 26°C water temperatures and across all temperatures combined. The arm fat mass, fat percentage and surface area to mass ratio were correlated with Tc cooling rates in most conditions but not all. Individuals in the high cooling rate group have approximately half the trunk fat mass of those in the low cooling group. Those with low trunk fat mass are least capable of defending body core temperature and should be considered most at risk for hypothermia when immersed in cool to cold water. The results suggest that trunk fat mass can potentially be used to identify individuals who are vulnerable to hypothermia risk during water immersion. Regional body composition provides additional body characteristics to study individual differences in thermal responses to cold water immersion.

## AUTHOR CONTRIBUTIONS

Xiaojiang Xu, conceptualization, data curation, formal analysis, methodology, project administration, writing – original draft, writing – review and editing; Stefan A. Gutierrez, formal analysis, methodology, writing – original draft, writing – review and editing; Timothy P. Rioux, formal analysis, methodology, project administration, writing – review and editing; Scott J. Montain, conceptualization, formal analysis, methodology, writing – original draft, writing – review and editing; David W. DeGroot, formal analysis, methodology, editing – review and editing; John W. Castellani, formal analysis, methodology, project administration, writing – original draft, writing – review and editing.

## FUNDING INFORMATION

This project was supported in part by a research fund from the Office of Naval Research.

## Data Availability

This study used third‐party data, and the author does not have permission to share. Requests to access the data should be directed to the third party and the author may provide contact information of the third party upon reasonable request.
